# Impairment of Hepcidin Upregulation by Lipopolysaccharide in the Interleukin-6 Knockout Mouse Brain

**DOI:** 10.3389/fnmol.2017.00367

**Published:** 2017-11-07

**Authors:** Fa-Li Zhang, Hui-Min Hou, Zhi-Nan Yin, Lan Chang, Fe-Mi Li, Y.-J. Chen, Ya Ke, Zhong-Ming Qian

**Affiliations:** ^1^Laboratory of Neuropharmacology, School of Pharmacy, Fudan University, Shanghai, China; ^2^National Pharmaceutical Engineering Research Center, Shanghai Institute of Pharmaceutical Industry, Shanghai, China; ^3^The First Affiliate Hospital, Biomedical Translational Research Institute, Guangdong Province Key Laboratory of Molecular Immunology and Antibody Engineering, Jinan University, Guangzhou, China; ^4^School of Biomedical Sciences and Gerald Choa Neuroscience Centre, Faculty of Medicine, The Chinese University of Hong Kong, Sha Tin, Hong Kong

**Keywords:** hepcidin, IL-6+/+ and IL-6-/- mice, signal transducer and activator of transcription 3 (STAT3), ferroportin 1 (Fpn1) and ferritin light chain (Ft-L), cortex and hippocampus, lipopolysaccharide (LPS)

## Abstract

To find out whether the Interleukin-6 (IL-6)/signal transducer and activator of transcription 3 (STAT3) signaling pathway is involved in the expression of hepcidin in the mouse brain *in vivo*, we investigated the phosphorylation of STAT3, as well as the expression of hepcidin mRNA, ferroportin 1 (Fpn1) and ferritin light chain (Ft-L) proteins in the cortex and hippocampus of LPS-treated wild type (IL-6+/+) and IL-6 knockout (IL-6-/-) mice. We demonstrated that IL-6 knockout could significantly reduce the response of hepcidin mRNA, phospho-STAT3, Fpn1 and Ft-L protein expression to LPS treatment, in both the cortex and hippocampus of mice. Also, Stattic, an inhibitor of STAT3, significantly reduced the expression of phospho-STAT3 and hepcidin mRNA in the cortex and hippocampus of the LPS-treated wild type mice. These findings provide *in vivo* evidence for the involvement of the IL-6/STAT3 signaling pathway in the expression of hepcidin.

## Introduction

Iron is the most abundant trace metal in the brain (Beard et al., 1993). The importance of iron for normal neurological function has been well-established. In the brain, neurons and glia require iron for many aspects of their physiology, including electron transport, myelination of axons, NADPH reductase activity, and as a co-factor for several enzymes involved in neurotransmitter synthesis (Benzi and Moretti, 1995; Gelman, 1995; Qian and Wang, 1998). However, iron is also a major generator of reactive oxygen species (Halliwell and Gutteridge, 1984). Abnormally high iron and iron-induced oxidative stress in the brain has been demonstrated to be an initial cause of neuronal death in neuroferritinopathy and aceruloplasminemia (Ke and Qian, 2003; Rouault, 2013; Arber et al., 2016), and also has been proposed to play a causative role in at least some of the other neurodegenerative diseases, including Alzheimer’s disease and Parkinson’s disease (Qian and Shen, 2001; Peters et al., 2015; Belaidi and Bush, 2016; Hare and Double, 2016).

Despite years of investigation, however, it is still not completely known why iron levels abnormally increase in certain regions of the brain in the patients with neurodegenerative disorders. Although it has been proposed that the disrupted expression or function of proteins involved in brain iron metabolism may be one of initial causes of abnormal iron accumulation in the brain (Qian and Wang, 1998; Qian and Shen, 2001), the reasons and mechanisms behind the induction of such a disruption are still not well-understood. Currently, we know very little about how the expression of iron transport proteins are controlled in the brain under physiological conditions. The understanding of this key question is essential for refining our understanding of brain iron metabolism, as well as of the reasons and mechanisms behind the disruption of brain iron metabolism in the development of neurodegenerative disorders.

In the periphery, it has been well-documented that hepcidin plays a central role in maintaining normal iron homeostasis by controlling the expression of iron transport proteins in the intestine, liver, spleen and bone marrow (Hentze et al., 2004; Nemeth and Ganz, 2006). This iron regulatory hormone is mainly synthesized by the liver, distributed in extracellular fluid, and excreted in urine (Krause et al., 2000; Park et al., 2001; Pigeon et al., 2001). Accumulated data have also shown that the peptide is homeostatically regulated by multiple signals, including iron stores, hypoxia, inflammation, and erythropoietic activity in the periphery (Nicolas et al., 2001; Wrighting and Andrews, 2006; Hentze et al., 2010; Ganz and Nemeth, 2011).

It has been confirmed that hepcidin is also widely expressed in the brain (Zechel et al., 2006; Wang et al., 2008; Hänninen et al., 2009). The peptide can regulate the expression of brain iron transport proteins, including its membrane receptor ferroportin 1 (Fpn1), implying a central role for the hormone in brain iron homeostasis (Raha-Chowdhury et al., 2015; Lu et al., 2016). However, information regarding the signals and mechanisms involved in the control of hepcidin expression in the brain, as opposed to within the periphery, is very limited. An *in vivo* study (Wang et al., 2010) demonstrated that lipopolysaccharide (LPS) could induce a significant increase in the expression of hepcidin in the cortex and substantia nigra of the rat brain, indicating that the expression of this iron regulatory hormone can also be regulated by inflammation as was found in the periphery.

Our recent *in vitro* study (Qian et al., 2014) demonstrated that LPS up-regulates the expression of hepcidin in neurons via microglia and the Interleukin-6 (IL-6)/signal transducer and activator of transcription 3 (STAT3) signaling pathway. In the present *in vivo* study, we compared changes in the phosphorylation of STAT3, the expression of hepcidin mRNA, ferroportin 1 (Fpn1) protein, and ferritin light chain (Ft-L) protein in the cortex and hippocampus of LPS-treated wild type (IL-6+/+) and IL-6 knockout (IL-6-/-) mice. We demonstrated that IL-6 KO could notably abolish the phosphorylation of STAT3 as well as expression of hepcidin mRNA, Fpn1 and Ft-L proteins in response to LPS treatment, in both the cortex and hippocampus of mice. Also, Stattic, an inhibitor of STAT3, was found to significantly reduce the expression of phospho-STAT3 and hepcidin mRNA in the cortex and hippocampus of LPS-treated wild type mice. Our findings provide further *in vivo* evidence for the involvement of the IL-6/STAT3 signaling pathway in the expression of hepcidin in the brain, under the conditions of inflammation.

## Materials and Methods

### Materials

Unless otherwise stated, all chemicals were obtained from the Sigma Chemical Company, St. Louis, MO, United States. Mouse anti-rat TfR1 (transferrin receptor 1) and fetal bovine serum (FBS) were purchased from Invitrogen, Carlsbad, CA, United States; rabbit polyclonal anti-mouse Fpn1 from Novus Biologicals, Littleton, CO, United States*;* rabbit polyclonal anti-Ft-L and anti-DMT1 (divalent metal transporter 1, SLC11A2) from Protein-tech, Chicago, IL, United States; and rabbit polyclonal anti-phospho-STAT3 (Tyr705) and mouse monoclonal anti-STAT3 both from Cell Signaling Technology, Inc., Danvers, MA, United States. Recombined mouse IL-6 protein was obtained from Gene Script, Piscataway, NJ, United States; IL-6 enzyme-linked immunosorbent assay (ELISA) kits from R&D Systems, Minneapolis, MN, United States; goat anti-rabbit or anti-mouse IRDye 800 CW secondary antibodies from LI-COR Bio Sciences, Lincoln, NE, United States; TRIzol reagent from Life Technologies, Carlsbad, CA, United States; AevertAid First Strand cDNA Synthesis Kit from Thermo Scientific, Waltham, MA, United States; FastStart Universal SYBR Green Master and LightCycler96 from Roche, Nutley, NJ, United States; and BCA protein Assay kit and protein RIPA lysis buffer from Beyotime Institute of Biotechnology, Haimen, JS, China.

### Mice

C57BL/6 male (IL-6+/+) mice (8-week-olds) were obtained from the Sippr-BK Experimental Animal Center, Shanghai, China. IL-6 knockout (KO) mice (IL-6-/-) mice were originally purchased from Jackson Laboratories, United States. The IL-6-/- and wild type mice were verified using RT-PCR (Supplementary Figure 1). Mice were housed in stainless steel cages at 21 ± 2°C with a relative humidity of 55–60% and alternating 12-h periods of light (07:00–19:00) and dark (19:00–07:00), with water and food supplied *ad libitum*. All animal care, surgical and experimental protocols were performed according to the Animal Management Rules of the Ministry of Health of China, and approved by the Animal Ethics Committees of Fudan University and The Chinese University of Hong Kong.

### Intracerebroventricular Injection of LPS

Intracerebroventricular (ICV) injection of LPS (*E. coli* serotype 055:B5) was performed as previously described (Liu et al., 2015; Gong et al., 2016). The mice were anesthetized with 4% chloral hydrate (1 ml/100 g body weight) via i.p. injection and were secured in a stereotaxic instrument. LPS (5 μg) in 2 μl phosphate buffered saline (PBS) or 2 μl endotoxin-free PBS (the control), were injected bilaterally into the lateral ventricle according to a standard stereotaxic atlas (-3.0 mm dorsal/ventral, -1.0 mm lateral, and -0.5 mm anterior/posterior from the bregma) using a 10 μl syringe with a 33 gauge needle at a rate of 0.5 μl/min. The syringe was left in place for an additional 5 min before removal. Tissue samples were harvested for analysis at 6- or 24-h after LPS injection.

### Enzyme-Linked Immunosorbent Assay

The concentrations of hepcidin and IL-6 were determined using ELISA kits according to suppliers’ instructions. Briefly, brain tissues were homogenized in PBS (1:5 w/v), followed by sonication using a Soniprep 150. The samples were centrifuged at 3,000 × *g* for 15 min at 4°C, and the supernatant was collected. A 2-μl aliquot was collected for detection of protein concentration. 100 μl of assay buffer and 50 μl of each standard, control, or sample were added into the appropriate wells, and 100 μl biotin conjugate was also dispensed into each well. Following incubation for 2-h at room temperature, the wells were washed with diluted wash solution, and 100 μl of enzyme complex was added to each well. After incubation for 1-h at room temperature, 100 μl substrate solution was added to each well. Finally, after being allowed to react for 30 min at room temperature, the enzymatic reaction was stopped by adding 100 μl of stop solution, and the optical density (OD) was read at 450 nm using an ELX-800 microplate assay reader (Bio-tek, United States). The average absorbance values for each set of standards, controls, and samples were calculated, and a standard curve was constructed. The concentrations of the samples were then calculated from the standard curve (Du et al., 2011; Qian et al., 2014).

### Isolation of Total RNA and Quantitative Real-time PCR

The extraction of total RNA and preparation of cDNA were performed using TRIzol reagent and the AevertAid First Strand cDNA Synthesis Kit respectively, in accordance with the instructions of the manufacturers. Real-time PCR was carried out by RT-PCR instrument (LC96, Roche, Switzerland) using Fast Start Universal SYBR Green Master and the Light Cycler96. The specific pairs of primers used were: mouse β-actin, forward: 5′-AAATCGTGCGTGACATCAAAGA-3′, reverse: 5′-GCCATCTCCTGCTCGAAGTC-3′; mouse hepcidin, forward: 5′-AGAGCTGCAGCCTTTGCAC-3′, reverse: 5′-GAAGATGCAGATGGGGAAGT-3′; and IL-6, forward: 5′-CTGCAAGAGACTTCCATCCAG-3′, reverse: 5′-AGTGGTATAGACAGGTCTGTTGG-3′ (Chang et al., 2006; Huang et al., 2014). The CT values of each target gene was normalized to that of the β-actin mRNA. Relative gene expression was calculated by the 2^-ΔΔC_T_^ method.

### Western Blot Analysis

The tissues were washed, homogenized by protein RIPA lysis buffer and then sonicated as described previously (Qian et al., 2011; Du et al., 2015). After centrifugation at 13200 rpm for 15 min at 4°C, the supernatant was collected and protein content was determined using the BCA protein Assay kit. Aliquots of the extract containing about 30 μg of protein were loaded and run on a single track of 10% SDS-PAGE under reducing conditions, and subsequently transferred to a pure nitrocellulose membrane (Bio-Rad). The blots were blocked in 5% non-fat milk and incubated with primary antibodies: mouse monoclonal anti-TfR1 (1:500), rabbit polyclonal anti-Fpn1 (1:1000), rabbit polyclonal anti-Ft-L (1:1000), rabbit polyclonal anti-DMT1 (1:1000) rabbit polyclonal anti-phospho-STAT3 (1:1000) and mouse monoclonal anti-STAT3 (1:1000). After being washed three times, the blots were incubated with goat anti-rabbit (1:1000) or anti-mouse IRDye800 CW secondary antibody (1:5000) for 2-h at room temperature. The intensities of the specific bands were detected and analyzed by an Odyssey infrared imaging system (Li-Cor, Lincoln, NE, United States). To ensure even loading of the samples, the same membrane was probed with mouse anti-β-actin polyclonal antibody at a 1:10,000 dilution (Ke et al., 2006; Du et al., 2010).

### Statistical Analysis

Statistical analyses were performed using Graphpad Prism. Data were presented as mean ± SEM. The differences between means were all determined by one-way or two-way analyses of variance (ANOVA) as appropriate, followed by Newman–Keuls *post hoc* tests. A probability value of *P* < 0.05 was taken to be statistically significant.

## Results

### LPS Induced a Significant Increase in the Expression of Hepcidin mRNA and IL-6 mRNA and Protein in the Cortex and Hippocampus of Wild Type Mice

**FIGURE 1:**
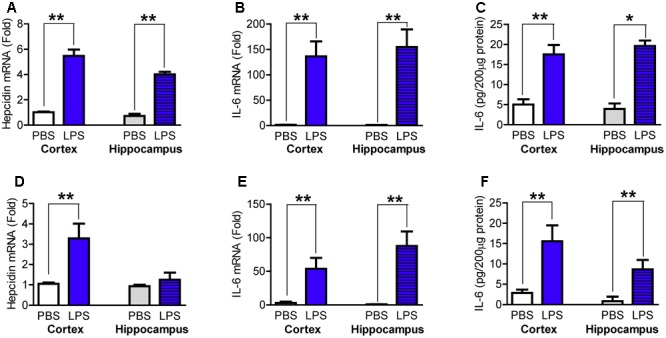
Lipopolysaccharide (LPS) induced a significant increase in the expression of hepcidin mRNA and IL-6 mRNA and protein in the cortex and hippocampus of wild type mice. Wild type mice were treated with LPS (5 μg) in 2 μl phosphate buffered saline (PBS) or 2 μl endotoxin-free PBS via ICV injection. At 6-h **(A–C)** and 24-h **(D,E)** after treatment, the expression of hepcidin **(A,D)** and IL-6 **(B,E)** mRNA in the cortex and hippocampus tissues were then measured by Quantitative Real-time PCR, and IL-6 protein **(C,F)** by ELISA as described in Section “Materials and Methods.” Data are means ± SEM (% Control) (*n*: 6-h: PBS = 4, LPS = 8; 24-h: PBS = 4, LPS = 6). ^∗^*P* < 0.05; ^∗∗^*P* < 0.01 vs. the mice injected with PBS.

Based on reported time-points in the response of hepcidin mRNA expression (Qian et al., 2014) and inflammatory cytokines (Lawson et al., 2013; Yanguas-Casás et al., 2014) to LPS treatment, we first measured hepcidin mRNA and IL-6 mRNA and protein content in the cortex and hippocampus of wild type mice at 6- and 24-h after LPS injection. It was found that at 6-h after LPS treatment, the expression of hepcidin mRNA (**Figure 1A**) and the expression of IL-6 mRNA (**Figure 1B**) and protein (**Figure 1C**) in the cortex and hippocampus were significantly higher in the LPS-treated mice than in the controls (PBS-treated mice). Hepcidin mRNA expression significantly increased in the cortex, but not the hippocampus (**Figure 1D**), and IL-6 mRNA (**Figure 1E**) and protein (**Figure 1F**) also increased in both the cortex and hippocampus in mice treated with LPS for 24-h, as compared with that of the control mice.

### Expression of Hepcidin mRNA in the Cortex and Hippocampus Was Significantly Lower in LPS-Treated IL-6 Knockout Mice than in LPS-Treated Wild Type Mice

**FIGURE 2:**
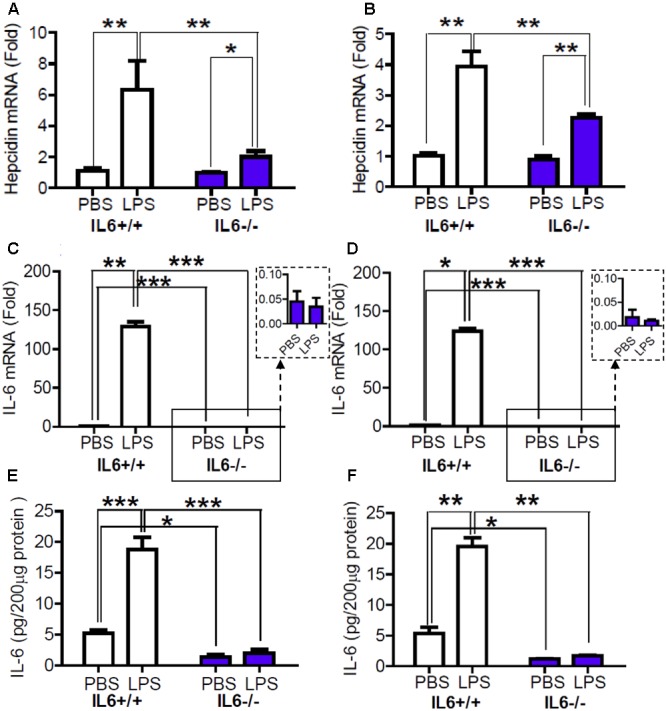
Expression of Hepcidin mRNA in the cortex and hippocampus was significantly lower in LPS-treated IL-6 knockout mice than in LPS-treated wild type mice. Wild type (IL-6+/+) and IL-6 knockout (IL-6–/–) mice were treated with LPS (5 μg) in 2 μl PBS or 2 μl endotoxin-free PBS (ICV injection) for 6-h. The expression of hepcidin **(A,B)** and IL-6 **(C,D)** mRNA was then measured by Quantitative Real-time PCR, and IL-6 protein **(E,F)** by ELISA in cortex **(A,C,E)** and hippocampus **(B,D,F)** tissue as described in Section “Materials and Methods.” Data are means ± SEM (% Control) (*n*: IL-6+/+ mice: PBS = 4, LPS = 6; IL-6–/– mice: PBS = 3, LPS = 4). ^∗^*P* < 0.05 ^∗∗^; *P* < 0.01; ^∗∗∗^*P* < 0.001 vs. the mice (IL-6+/+ or IL-6–/–) injected with PBS or LPS.

To confirm the role of IL-6 in hepcidin expression’s response to LPS treatment, we then investigated the effects of LPS treatment on the expression of hepcidin mRNA in IL-6 knockout (IL-6-/-) mice and wild type (IL-6+/+) mice. RT-PCR analysis showed that there were no significant differences in the expression of hepcidin mRNA in the cortex (**Figure 2A**) and hippocampus (**Figure 2B**) between the IL-6+/+ and the IL-6-/- mice, both treated with PBS. However, LPS treatment induced a significant increase in the expression of hepcidin mRNA in these two brain regions in the IL-6+/+ mice, being about sixfold (cortex) and fourfold (hippocampus) of the controls. LPS treatment also led to a significant increase in the expression of hepcidin mRNA in the cortex (**Figure 2A**) and hippocampus (**Figure 2B**) of the IL-6-/- mice, being about twofold (cortex) and 2.5-fold (hippocampus) of the controls. The expression of hepcidin mRNA in the cortex (**Figure 2A**) and hippocampus (**Figure 2B**) in the LPS-treated IL-6-/- mice was significantly lower than in the LPS-treated IL-6+/+ mice. These findings indicated that IL-6-/- largely reduced the expression of hepcidin in response to LPS treatment. In addition, RT-PCR and Western analysis demonstrated that the expression of IL-6 mRNA and protein in the cortex (**Figures 2C,E**) and hippocampus (**Figures 2D,F**) was significantly lower in IL-6-/- mice than in the controls (IL-6+/+). There were no significant differences in these measurements between the LPS- or PBS-treated IL-6-/- mice.

### pSTAT3/STAT3 in the Cortex and Hippocampus Was Significantly Lower in LPS-Treated IL-6 Knockout Mice than in LPS-Treated Wild Type Mice

**FIGURE 3:**
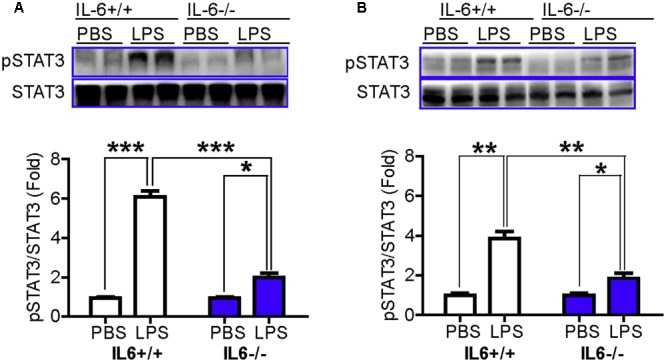
pSTAT3/STAT3 in the cortex and hippocampus was significantly lower in LPS-treated IL-6 knockout mice than in LPS-treated wild type mice. Wild type (IL-6+/+) and IL-6 knockout (IL-6–/–) mice were treated with LPS (5 μg) in 2 μl PBS or 2 μl endotoxin-free PBS via ICV injection. At 6-h after treatment, pSTAT3 and STAT3 contents in cortex **(A)** and hippocampus **(B)** tissue were then measured by western blot analysis as described in Section “Materials and Methods.” Data are means ± SEM (% Control) (*n*: IL-6+/+ mice: PBS = 3, LPS = 6; IL-6–/– mice: PBS = 3, LPS = 3). ^∗^*P* < 0.05; ^∗∗^*P* < 0.01; ^∗∗∗^*P* < 0.001 vs. the mice (IL-6+/+ or IL-6–/–) injected with PBS or LPS.

To further clarify the involvement of STAT3 in LPS-induced up-regulation of hepcidin (Qian et al., 2014), we then examined the effects of IL-6-/- on the phosphorylation of STAT3 in mice. It was found that there were no significant differences in pSTAT3/STAT3 levels in the cortex (**Figure 3A**) and hippocampus (**Figure 3B**) between IL-6+/+ and IL-6-/- mice, both treated with PBS. However, LPS treatment was found to induce a significant increase in the levels of pSTAT3/STAT3 in these two brain regions in wild type mice, to about sixfold (cortex) and fourfold (hippocampus) of the controls. LPS treatment also led to a significant increase in the level of pSTAT3/STAT3 in the cortex (**Figure 3A**) and hippocampus (**Figure 3B**) of the IL-6-/- mice, both being about twofold of the controls. Again, pSTAT3/STAT3 levels in the cortex (**Figure 3A**) and hippocampus (**Figure 3B**) in the LPS-treated IL-6-/- mice was found to be significantly lower than those of the LPS-treated IL-6+/+ mice. The data imply that IL-6 KO can significantly abolish not only the response of hepcidin mRNA expression to LPS treatment, but also the response of phosphorylation of STAT3.

### IL-6 Deficiency Abolished the LPS-Induced Reduction in the Expression of Ferroportin 1 and the Increase in Ferritin Light Chain Protein Levels in Mice

**FIGURE 4:**
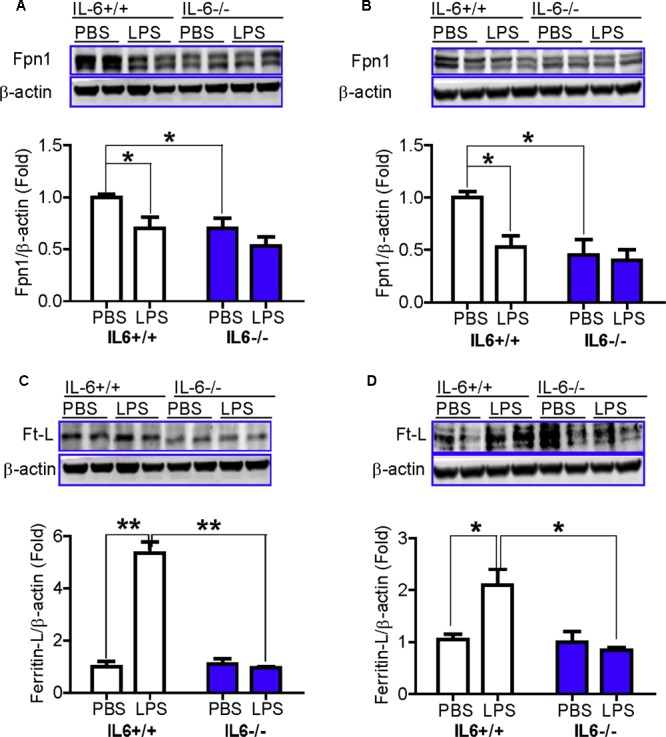
IL-6 deficiency abolished the LPS-induced reduction in the expression of ferroportin 1 and the increase in ferritin light chain protein levels in mice. Wild type (IL-6+/+) and IL-6 knockout (IL-6–/–) mice were treated with LPS (5 μg) in 2 μl PBS or 2 μl endotoxin-free PBS via ICV injection. At 6-h after treatment, Fpn1 and Ft-L proteins in cortex **(A,C)** and hippocampus **(B,D)** tissue were then measured by western blot analysis as described in Section “Materials and Methods.” Data are means ± SEM (% Control) (*n*: IL-6+/+ mice: PBS = 3, LPS = 6; IL-6–/– mice: PBS = 3, LPS = 3). ^∗^*P* < 0.05; ^∗∗^*P* < 0.01 vs. the mice (IL-6+/+ or IL-6–/–) injected with PBS or LPS.

**FIGURE 5:**
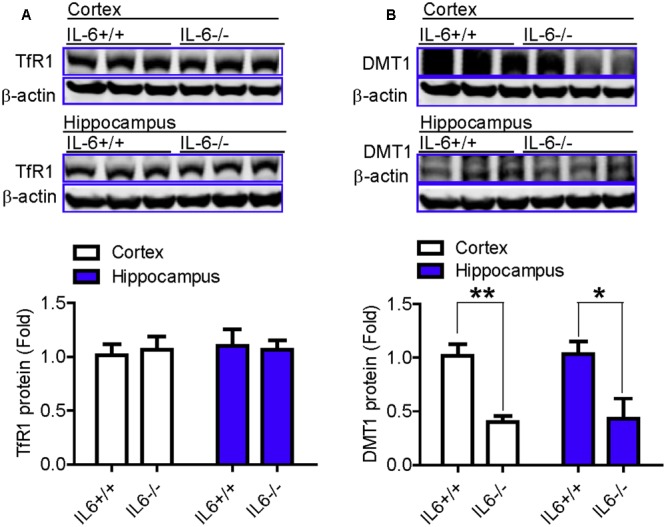
The expression of DMT1 in the brain was significantly lower in the IL-6–/– mice than in the IL-6+/+ mice. Wild type (IL-6+/+) and IL-6 knockout (IL-6–/–) mice were treated with LPS (5 μg) in 2 μl PBS or 2 μl endotoxin-free PBS via ICV injection. At 6-h after treatment, TfR1 **(A)** and DMT1 **(B)** proteins in cortex and hippocampus tissue were then measured by western blot analysis as described in Section “Materials and Methods.” Data are means ± SEM (% Control) (*n*: IL-6+/+ mice: PBS = 3, LPS = 6; IL-6–/– mice: PBS = 3, LPS = 3). ^∗^*P* < 0.05; ^∗∗^*P* < 0.01 vs. the mice (IL-6+/+ or IL-6–/–) injected with PBS.

We also examined changes in the expression of Fpn1 and in Ft-L (ferritin light chain) content in the cortex and hippocampus of wild type and IL-6 KO mice, treated with or without LPS. The expression of Fpn1 and Ft-L was investigated, because Fpn1 is membrane receptor of hepcidin (Nemeth et al., 2004; Qiao et al., 2012) while Ft-L is a marker of cell-iron content, which can be affected by changes in Fpn1 expression (Du et al., 2012). We found that LPS treatment induced a significant reduction in the expression of Fpn1 (**Figures 4A,B**) and an increase in Ft-L content (**Figures 4C,D**), in both the cortex and hippocampus of IL-6+/+ mice, while such a change was not found in the IL-6-/- mice (**Figures 4A–D**). This implies that IL-6 deficiency can significantly abolish the LPS-induced reduction of Fpn 1 expression and increase in Ft-L protein levels in mice. While the expression of Fpn1 was lower in the cortex and hippocampus of LPS-treated IL-6-/- mice than in the PBS-treated IL-6-/- mice, the difference was not significant. Also, Ft-L contents in these two brain regions in the LPS-treated IL-6-/- mice did not differ from those in the PBS-treated IL-6-/- mice. In addition, western blot analysis showed that Fpn1 expression in both the cortex (**Figure 4A**) and hippocampus (**Figure 4B**) of wild type mice were significantly higher than in those of the IL-6-/- mice (about 1.5–2 fold). The expression of DMT1 (divalent metal transporter 1) in both the cortex (**Figure 5B**) and hippocampus (**Figure 5B**) was also found to be significantly lower in the IL-6-/- mice than in the IL-6+/+ mice. There were no differences in TfR1 (transferrin receptor 1) protein content in the brains of IL-6-/- and IL-6+/+ mice (**Figure 5A**).

### Stattic Significantly Reduced the Phosphorylation of STAT3 and the Expression of Hepcidin mRNA in Mice Treated with LPS

**FIGURE 6:**
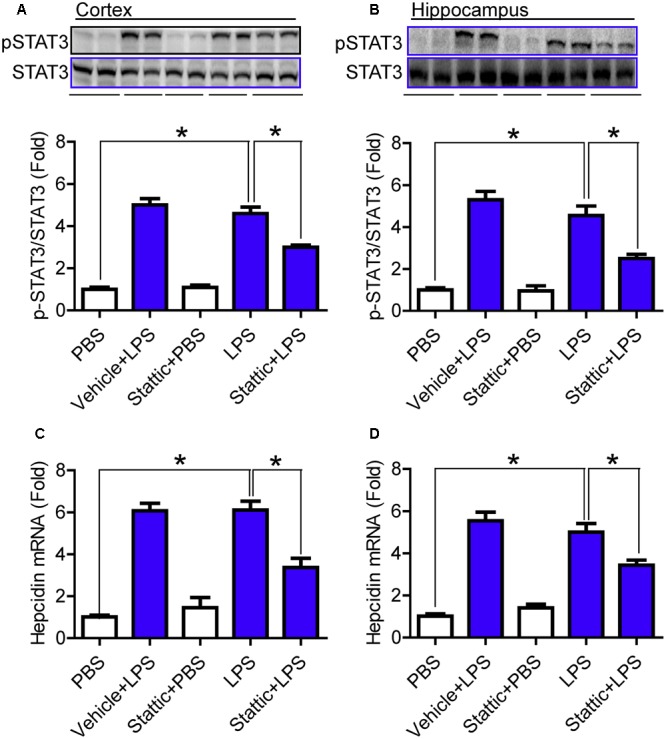
Stattic significantly reduced the phosphorylation of STAT3 and the expression of hepcidin mRNA in mice treated with LPS. C57BL6 male mice were pre-treated with 20 mg/kg Stattic (an inhibitor of STAT3) in 2% DMSO with 30% PEG300 (IP injection) or vehicle (control) for 30 min and then with 5 μg LPS in 2 μl PBS or PBS (ICV injection) for 6-h. Phosphorylation of STAT3 **(A,B)** and expression of hepcidin mRNA **(C,D)** in the cortex **(A,C)** and hippocampus **(B,D)** were measured by western blot analysis or RT-PCR analysis as described in Section “Materials and Methods.” Data are means ± SEM (% Control) (*n* = 4) ^∗^*P* < 0.05 vs. the control mice.

Finally, we investigated the effects of Stattic (an inhibitor of STAT3) on the phosphorylation of STAT3 and the expression of hepcidin mRNA in the cortex and hippocampus of mice treated with LPS, in order to confirm the involvement of STAT3 activation in the expression of hepcidin. C57BL6 male mice (8-week-olds) were pre-treated with 20 mg/kg Stattic (Sigma-Aldrich) in 2% DMSO with 30% PEG300 (i.p. injection) or vehicle (control) for 30 min and then with 5 μg LPS in 2 μl sterile saline (i.c.v. injection) for 6-h. It was found that the levels of pSTAT3 (**Figures 6A,B**) and hepcidin mRNA (**Figures 6C,D**) were significantly lower in the cortex (**Figures 6A,C**) and hippocampus (**Figures 6B,D**) of mice pre-treated with Stattic than of those in the control mice, indicating that inhibiting STAT3 with Stattic can significantly reduce the phosphorylation of STAT3 and the expression of hepcidin mRNA in mice treated with LPS.

## Discussion

A recent study we published using a co-culture model of neurons with microglia *in vitro*, showed that neurons are the major cells of the increased hepcidin expression that occurs in response to LPS challenge, although microglia play a key mediating role as well, by releasing IL-6 and recruiting the STAT3 pathway, indicating that LPS up-regulates hepcidin expression in neurons via microglia and the IL-6/STAT3 signaling pathway (Qian et al., 2014). In the present study, we demonstrate that the levels of phosphorylated STAT3 and hepcidin mRNA content in the cortex and hippocampus of IL-6-/- mice are not different to those of IL-6+/+ mice. However, under the conditions of LPS treatment, these two indices were found to be significantly lower in the IL-6-/- mice than of those in the IL-6+/+ mice. The results show that IL-6 KO significantly reduces the response of STAT3 phosphorylation and hepcidin expression to LPS challenge in these two brain regionsin mice. Also, we found that pre-treatment with Stattic (an inhibitor of STAT3) significantly reduced phosphorylation of STAT3 and expression of hepcidin mRNA in the cortex and hippocampus of mice treated with LPS. These findings provided solid *in vivo* evidence for the involvement of the IL-6/STAT3 signaling pathway in the expression of hepcidin in the brain.

pSTAT3/STAT3 and hepcidin mRNA levels in the cortex and hippocampus of the IL-6-/- mice were significantly lower compared to those of the IL-6+/+ mice under the conditions of LPS treatment, although these two measurements were still found to be higher in LPS-treated IL-6-/- mice than those in PBS-treated IL-6-/- mice. This indicated that phosphorylation of STAT3 and expression of hepcidin in the brain still have the ability to respond to LPS treatment, although this ability was markedly abolished by IL-6 KO under our experimental conditions. Increased phosphorylation of STAT3 and expression of hepcidin increase the binding of hepcidin with its membrane receptor Fpn1, leading to the internalization and degradation of the Hepcidin/Fpn1 complex (Nemeth et al., 2004; Nemeth and Ganz, 2006; Wrighting and Andrews, 2006; Hentze et al., 2010; Ganz and Nemeth, 2011). Therefore, although the difference was not significant, the increased expression of hepcidin was likely to be one of the causes for the reduction in Fpn1 expression found in both the cortex and hippocampus of the LPS-treated IL-6-/- mice, as compared with the PBS-treated IL-6-/- mice.

The expression of Fpn1 in the cortex and hippocampus of the IL-6-/- mice treated with PBS was also found to be significantly lower than that of the IL-6+/+ mice treated with PBS (the control). The reduction in the expression of Fpn1 is unlikely to be associated with the phosphorylation of STAT3 and the expression of hepcidin, because the expression of pSTAT3 and hepcidin in both the cortex and hippocampus of the IL-6-/- mice treated with PBS did not differ from those of the IL-6+/+ mice treated without LPS (the control). It may be the case that the IL-6 KO itself has a role in inhibiting the expression of Fpn1, although the relevant mechanisms are currently unknown.

In addition to the iron release protein Fpn1, iron content in cells or tissues also depend on the expression of iron uptake proteins such as TfR1 and DMT1 (Qian and Ke, 2001; Jiang et al., 2002). TfR1-mediated transferrin-bound iron (Tf-fe) uptake is the main route for mammalian cellular iron accumulation, while. DMT1 is involved in the translocation of iron from the endosome to the cytosol, being essential for Tf-bound iron uptake under physiological conditions (Qian et al., 1997; Ke and Qian, 2007). Therefore, the significant reduction in DMT1 expression, induced by IL-6 KO, might partly be associated with the un-changed Ft-L content found in the two brain regions investigated in the IL-6-/- mice under conditions of LPS treatment.

In the present study, we also found that in IL-6-/- mice, IL-6 mRNA and protein levels were almost totally abolished, although LPS treatment could still induce an increase in the phosphorylation of STAT3 and in the expression of hepcidin mRNA, as well as a reduction in Fpn1 content in the cortex and hippocampus. This implies that other molecules or inflammatory cytokines, in addition to IL-6, may also have a role in regulating hepcidin expression. These molecules may include IL-22 and IL-1, and the involvement of these molecules in the up-regulation of STAT3 phosphorylation and/or hepcidin expression has been reported (Lee et al., 2005; Armitage et al., 2011; Ryan et al., 2012). Also, it has recently been reported that interleukin 1β (IL-1β) up-regulates the expression of hepcidin by activating the bone morphogenetic protein (BMP) signaling pathway (Shanmugam et al., 2015) or inducing CCAAT enhancer-binding protein δ (C/EBPδ) expression (Kanamori et al., 2017). Based on the significant effect of IL-6-/- on the phosphorylation of STAT3 and/or the expression of hepcidin found in the present study, it may be reasonable however, to infer that IL-6 plays a predominant role in controlling hepcidin expression in the brain under the conditions of inflammation and infection.

In the rat brain, LPS treatment was also found to induce a significant increase in the expression of hepcidin mRNA and protein in the cortex and substantia nigra, though not in the hippocampus and striatum (Wang et al., 2008). There were no significant differences in the expression of hepcidin mRNA and protein between the control values and all measurements taken at different time points after LPS administration in the hippocampus and striatum (Wang et al., 2008). This finding indicates that the response of hepcidin expression to LPS may differ by region in the rat brain. In the present study, LPS treatment for 6-h was demonstrated to induce a significant increase in IL-6 mRNA expression, STAT3 phosphorylation and hepcidin mRNA expression, not only in the cortex but also in the hippocampus in the mouse brain. This implies that the response of hepcidin expression to LPS may also differ by species in the brain. The regional specificity observed may be partly associated with the abundance of microglia in different brain regions (Lawson et al., 1990; Kim et al., 2000), while the reasons responsible for the interspecies difference are currently unknown; further investigations are needed.

## Author Contributions

H-MH, YK, and Z-MQ conceived, organized and supervised the study; F-LZ, F-ML, Y-JC, and LC performed the experiments; H-MH, Z-NY, and YK contributed to the analysis of data, H-MH, YK, and Z-MQ prepared and wrote the manuscript.

## Conflict of Interest Statement

The authors declare that the research was conducted in the absence of any commercial or financial relationships that could be construed as a potential conflict of interest.

## References

[B1] Arber C. E. Li A. Houlden H. Wray S. (2016). Review: insights into molecular mechanisms of disease in neurodegeneration with brain iron accumulation: unifying theories. *Neuropathol. Appl. Neurobiol.* 42 220–241. 10.1111/nan.12242 25870938 PMC4832581

[B2] Armitage A. E. Eddowes L. A. Gileadi U. Cole S. Spottiswoode N. Selvakumar T. A. (2011). Hepcidin regulation by innate immune and infectious stimuli. *Blood* 118 4129–4139. 10.1182/blood-2011-04-351957 21873546

[B3] Beard J. L. Connor J. R. Jones B. C. (1993). Iron in the brain. *Nutr. Rev.* 52 157–170. 10.1111/j.1753-4887.1993.tb03096.x 8371846

[B4] Belaidi A. A. Bush A. I. (2016). Iron neurochemistry in Alzheimer’s disease and Parkinson’s disease: targets for therapeutics. *J. Neurochem.* 139 179–197. 10.1111/jnc.13425 26545340

[B5] Benzi G. Moretti A. (1995). Are reactive oxygen species involved in Alzheimer’s disease? *Neurobiol. Aging* 16 661–674. 10.1371/journal.pntd.0005539 8544918

[B6] Chang Y. Z. Ke Y. Du J. R. Halpern G. M. Ho K. P. Zhu L. (2006). Increased divalent metal transporter 1 expression might be associated with the neurotoxicity of L-DOPA. *Mol. Pharmacol.* 69 968–974. 16317110 10.1124/mol.105.017756

[B7] Du F. Qian C. Qian Z. M. Wu X. M. Xie H. Yung W. H. (2011). Hepcidin directly inhibits transferrin receptor 1 expression in astrocytes via a cyclic AMP-protein kinase A pathway. *Glia* 59 936–945. 10.1002/glia.21166 21438013

[B8] Du F. Qian Z. M. Gong Q. Zhu Z. J. Lu L. Ke Y. (2012). The iron regulatory hormone hepcidin inhibits expression of iron release as well as iron uptake proteins in J774 cells. *J. Nutr. Biochem.* 23 1694–1700. 10.1016/j.jnutbio.2011.12.002 22560353

[B9] Du F. Qian Z. M. Luo Q. Yung W. H. Ke Y. (2015). Hepcidin suppresses brain iron accumulation by downregulating iron transport proteins in iron-overloaded rats. *Mol. Neurobiol.* 52 101–114. 10.1007/s12035-014-8847-x 25115800

[B10] Du F. Qian Z. M. Zhu L. Wu X. M. Qian C. Chan R. (2010). Purity, cell viability, expression of GFAP and bystin in astrocytes cultured by different procedures. *J. Cell. Biochem.* 109 30–37. 19899109 10.1002/jcb.22375

[B11] Ganz T. Nemeth E. (2011). Hepcidin and disorders of ironmetabolism. *Annu. Rev. Med.* 62 347–360. 10.1146/annurev-med-050109-142444 20887198

[B12] Gelman B. B. (1995). Iron in CNS disease. *J. Neuropathol. Exp. Neurol.* 54 477–486. 10.1097/00005072-199507000-00001 7602322

[B13] Gong J. Du F. Qian Z. M. Luo Q. Q. Sheng Y. Yung W. H. (2016). Pre-treatment of rats with ad-hepcidin prevents iron-induced oxidative stress in the brain. *Free Radic. Biol. Med.* 90 126–132. 10.1016/j.freeradbiomed.2015.11.016 26582371

[B14] Halliwell B. Gutteridge J. M. (1984). Oxygen toxicity, oxygen radicals, transition metals and disease. *Biochem. J.* 219 1–14. 10.1042/bj2190001 6326753 PMC1153442

[B15] Hänninen M. M. Haapasalo J. Haapasalo H. Fleming R. E. Britton R. S. Bacon B. R. (2009). Expression of iron-related genes in human brain and brain tumors. *BMC Neurosci.* 10:36. 10.1186/1471-2202-10-36 19386095 PMC2679039

[B16] Hare D. J. Double K. L. (2016). Iron and dopamine: a toxic couple. *Brain* 139 1026–1035. 10.1093/brain/aww022 26962053

[B17] Hentze M. W. Muckenthale M. U. Andrews N. C. (2004). Balancing acts: molecular control of mammalian iron metabolism. *Cell* 117 285–297. 10.1016/S0092-8674(04)00343-5 15109490

[B18] Hentze M. W. Muckenthaler M. U. Galy B. Camaschella C. (2010). Two to tango: regulation of mammalian iron metabolism. *Cell* 142 24–38. 10.1016/j.cell.2010.06.028 20603012

[B19] Huang X. T. Qian Z. M. He X. Gong Q. Wu K. C. Jiang L. R. (2014). Reducing iron in the brain: a novel pharmacologic mechanism of huperzine A in the treatment of Alzheimer’s disease. *Neurobiol. Aging* 35 1045–1054. 10.1016/j.neurobiolaging.2013.11.004 24332448

[B20] Jiang D. H. Ke Y. Cheng Y. Z. Ho K. P. Qian Z. M. (2002). Distribution of ferroportin1 protein in different regions of developing rat brain. *Dev. Neurosci.* 24 94–98. 10.1159/000065687 12401946

[B21] Kanamori Y. Murakami M. Sugiyama M. Hashimoto O. Matsui T. Funaba M. (2017). Interleukin-1β (IL-1β) transcriptionally activates hepcidin by inducing CCAAT enhancer-binding protein δ (C/EBPδ) expression in hepatocytes. *J. Biol. Chem.* 292 10275–10287. 10.1074/jbc.M116.770974 28438835 PMC5473230

[B22] Ke Y. Ho K. Du J. Zhu L. Xu Y. Wang Q. (2006). Role of soluble ceruloplasmin in iron uptake by midbrain and hippocampus neurons. *J. Cell. Biochem.* 98 912–919. 10.1002/jcb.20740 16475160

[B23] Ke Y. Qian Z. M. (2003). Iron misregulation in the brain: a primary cause of neurodegenerative disorders. *Lancet Neurol.* 2 246–253. 10.1016/S1474-4422(03)00353-3 12849213

[B24] Ke Y. Qian Z. M. (2007). Brain iron metabolism: neurobiology and neurochemistry. *Prog. Neurobiol.* 83 149–173. 10.1016/j.pneurobio.2007.07.009 17870230

[B25] Kim W. G. Mohney R. P. Wilson B. Jeohn G. H. Liu B. Hong J. S. (2000). Regional difference in susceptibility to lipopolysaccharide-induced neurotoxicity in the rat brain: role of microglia. *J. Neurosci.* 20 6309–6316. 10934283 10.1523/JNEUROSCI.20-16-06309.2000 PMC6772569

[B26] Krause A. Neitz S. Magrert H. J. Schulz A. Forssmann W. G. Schulz-Knappe P. (2000). LEAP-1, a novel highly disulfide-bonded human peptide, exhibits antimicrobial activity. *FEBS Lett.* 480 147–150. 10.1016/S0014-5793(00)01920-7 11034317

[B27] Lawson L. J. Perry V. H. Dri P. Gordon S. (1990). Heterogeneity in the distribution and morphology of microglia in the normal adult mouse brain. *Neuroscience* 39 151–170. 10.1016/0306-4522(90)90229-W 2089275

[B28] Lawson M. A. McCusker R. H. Kelley K. W. (2013). Interleukin-1 beta converting enzyme is necessary for development of depression-like behavior following intracerebroventricular administration of lipopolysaccharide to mice. *J. Neuroinflammation* 10:54. 10.1186/1742-2094-10-54 23634700 PMC3663735

[B29] Lee P. Peng H. Gelbart T. Wang L. Beutler E. (2005). Regulation of hepcidin transcription by interleukin-1 and interleukin-6. *Proc. Natl. Acad. Sci. U.S.A.* 102 1906–1910. 10.1073/pnas.0409808102 15684062 PMC548537

[B30] Liu Y. Wu X. M. Luo Q. Q. Huang S. Yang Q. W. Wang F. X. (2015). CX3CL1/CX3CR1-mediated microglia activation plays a detrimental role in ischemic mice brain via p38MAPK/PKC pathway. *J. Cereb. Blood Flow. Metab.* 35 1623–1631. 10.1038/jcbfm.2015.97 25966946 PMC4640309

[B31] Lu L. N. Qian Z. M. Wu K. C. Yung W. H. Ke Y. (2016). Expression of iron transporters and pathological hallmarks of Parkinson’s and Alzheimer’s diseases in the brain of young, adult, and aged rats. *Mol. Neurobiol.* 54 5213–5224. 10.1007/s12035-016-0067-0 27578012

[B32] Nemeth E. Ganz T. (2006). Regulation of iron metabolism by hepcidin. *Annu. Rev. Nutr.* 26 323–342. 10.1146/annurev.nutr.26.061505.111303 16848710

[B33] Nemeth E. Tuttle M. S. Powelson J. (2004). Hepcidin regulates cellular iron efflux by binding to ferroportin and inducing its internalization. *Science* 306 2090–2093. 10.1126/science.1104742 15514116

[B34] Nicolas G. Bennoun M. Devaux I. Beaumont C. Grandchamp B. Kahn A. (2001). Lack of hepcidin gene expression and severe tissue iron overload in upstream stimulatory factor 2 (USF2)knockout mice. *Proc. Natl. Acad. Sci. U.S.A.* 98 8780–8785. 10.1073/pnas.151179498 11447267 PMC37512

[B35] Park C. H. Valore E. V. Waring A. J. Ganz T. (2001). Hepcidin, a urinary antimicrobial peptide synthesized in the liver. *J. Biol. Chem.* 276 7806–7810. 10.1074/jbc.M008922200 11113131

[B36] Peters D. G. Connor J. R. Meadowcroft M. D. (2015). The relationship between iron dyshomeostasis and amyloidogenesis in Alzheimer’s disease: two sides of the same coin. *Neurobiol. Dis.* 81 49–65. 10.1016/j.nbd.2015.08.007 26303889 PMC4672943

[B37] Pigeon C. Ilyin G. Courselaud B. Leroyer P. Turlin B. Brissot P. (2001). A newmouse liver-specific gene, encoding a protein homologous to human antimicrobial peptide hepcidin, is overexpressed during iron overload. *J. Biol. Chem.* 276 7811–7819. 10.1074/jbc.M008923200 11113132

[B38] Qian Z. M. He X. Liang T. Wu K. C. Yan Y. C. Lu L. N. (2014). Lipopolysaccharides upregulate hepcidin in neuron via microglia and the IL-6/STAT3 signaling pathway. *Mol. Neurobiol.* 50 811–820. 10.1007/s12035-014-8671-3 24659348

[B39] Qian Z. M. Ke Y. (2001). Rethinking the role of ceruloplasmin in brain iron metabolism. *Brain Res. Rev.* 35 287–294. 10.1016/S0165-0173(01)00056-X 11423158

[B40] Qian Z. M. Shen X. (2001). Brain iron transport and neurodegeneration. *Trends Mol. Med.* 7 103–108. 10.1016/S1471-4914(00)01910-9 11286780

[B41] Qian Z. M. Tang P. L. Wang Q. (1997). Iron crosses the endosomal membrane by a carrier-mediated process. *Prog. Biophys. Mol. Biol.* 67 1–15. 10.1016/S0079-6107(97)00009-6 9401416

[B42] Qian Z. M. Wang Q. (1998). Expression of iron transport proteins and excessive iron accumulation in the brain in neurodegenerative disorders. *Brain Res. Rev.* 27 257–267. 10.1016/S0165-0173(98)00012-5 9729418

[B43] Qian Z. M. Wu X. M. Fan M. Yang L. Du F. Yung W. H. (2011). Divalent metal transporter 1 is a hypoxia-inducible gene. *J. Cell. Physiol.* 226 1596–1603. 10.1002/jcp.22485 20945371

[B44] Qiao B. Sugianto P. Fung E. (2012). Hepcidin-induced endocytosis of ferroportin is dependent on ferroportin ubiquitination. *Cell Metab.* 15 918–924. 10.1016/j.cmet.2012.03.018 22682227 PMC3372862

[B45] Raha-Chowdhury R. Raha A. A. Forostyak S. Zhao J. W. Stott S. R. Bomford A. (2015). Expression and cellular localization of hepcidin mRNA and protein in normal rat brain. *BMC Neurosci.* 16:24. 10.1186/s12868-015-0161-7 25896789 PMC4409766

[B46] Rouault T. A. (2013). Iron metabolism in the CNS: implications for neurodegenerative diseases. *Nat. Rev. Neurosci.* 14 551–564. 10.1038/nrn3453 23820773

[B47] Ryan J. D. Altamura S. Devitt E. Mullins S. Lawless M. W. Muckenthaler M. U. (2012). Pegylated interferon-α induced hypoferremia is associated with the immediate response to treatment in hepatitis C. *Hepatology* 56 492–500. 10.1002/hep.25666 22334511

[B48] Shanmugam N. K. Chen K. Cherayil B. J. (2015). Commensal Bacteria-induced Interleukin 1β (IL-1β) secreted by macrophages up-regulates hepcidin expression in hepatocytes by activating the bone morphogenetic protein signaling pathway. *J. Biol. Chem.* 290 30637–30647. 10.1074/jbc.M115.689190 26515063 PMC4683283

[B49] Wang Q. Du F. Qian Z. M. Ge X. H. Zhu L. Yung W. H. (2008). Lipopolysaccharide induces a significant increase in expression of iron regulatory hormone hepcidin in the cortex and substantia nigra in rat brain. *Endocrinology* 149 3920–3925. 10.1210/en.2007-1626 18450970 PMC2488231

[B50] Wang S. M. Fu L. J. Duan X. L. Crooks D. R. Yu P. Qian Z. M. (2010). Role of hepcidin in murine brain iron metabolism. *Cell. Mol. Life Sci.* 67 123–133. 10.1007/s00018-009-0167-3 19898775 PMC4225129

[B51] Wrighting D. M. Andrews N. C. (2006). Interleukin-6 induces hepcidin expression through STAT3. *Blood* 108 3204–3209. 10.1182/blood-2006-06-027631 16835372 PMC1895528

[B52] Yanguas-Casás N. Barreda-Manso M. A. Nieto-Sampedro M. (2014). Tauroursodeoxycholic acid reduces glial cell activation in an animal model of acute neuroinflammation. *J. Neuroinflammation* 11:50. 10.1186/1742-2094-11-50 24645669 PMC4000131

[B53] Zechel S. Huber-Wittmer K. von Bohlen Und Halbach O. (2006). Distribution of the iron-regulating protein hepcidin in the murine central nervous system. *J. Neurosci. Res.* 84 790–800. 10.1002/jnr.20991 16933319

