# Supplementary material for: Impairment of Hepcidin Upregulation by Lipopolysaccharide in the Interleukin-6 Knockout Mouse Brain
Source: Front Mol Neurosci. 2017 Nov 7;10:367. doi: 10.3389/fnmol.2017.00367 (PMC5681933; doi:10.3389/fnmol.2017.00367)

Supplementary Figure1

Targeted disruption of the mouse IL-6 gene.

Genotyping on mouse-tail DNA was performed by using a single PCR reaction to identify wild-type and IL-6 knockout mice. PCR-genotyping of IL-6 knockout mice was performed using a three-primer assay in one reaction. Forward: TTCCATCCAGTTGCCTTCTTGG, Reverse 1 : TTCTCATTTCCACGATTTCCCAG, Reverse 2 : CCGGAGAACCTGCGTGCAATCC,as described previously by Hilbert et al in 1995 (Hilbert DM, Kopf M, Mock BA, Köhler G, Rudikoff S. Interleukin 6 is essential for in vivo development of B lineage neoplasms. J Exp Med. 1995 Jul 1;182(1):243-248). The IL-6-/- mice product size is 380bp, the IL-6+/+ mice product size is 174bp, the IL-6+/- mice product size is 380bp and 174bp.

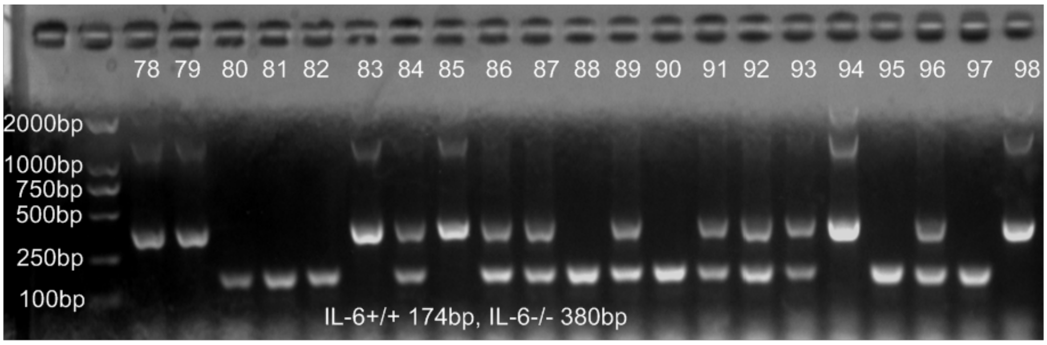

Supplement: Supplementary file 1 [file Image_1.PDF]
